# A Label-Free, Quantitative Assay of Amyloid Fibril Growth Based on Intrinsic Fluorescence

**DOI:** 10.1002/cbic.201300103

**Published:** 2013-04-16

**Authors:** Dorothea Pinotsi, Alexander K Buell, Christopher M Dobson, Gabriele S Kaminski Schierle, Clemens F Kaminski

**Affiliations:** [a] Department of Chemical Engineering and Biotechnology, University of CambridgePembroke Street, Cambridge, CB2 3RA (UK); [b] Department of Chemistry, University of CambridgeLensfield Road, Cambridge, CB2 1EW (UK).

**Keywords:** amyloid fibrils, intrinsic fluorescence, kinetic assays, protein aggregation

## Introduction

Unraveling the fundamental mechanisms and pathways behind protein misfolding and aggregation is one of the great challenges of current science. The failure of specific proteins to fold correctly or to remain correctly folded and their subsequent self-assembly into dysfunctional or indeed toxic aggregates is associated with a range of highly debilitating and increasingly prevalent pathological conditions.[1] These include neurodegenerative disorders, such as Alzheimer’s (AD) and Parkinson’s (PD) diseases, in which the peptide fragment amyloid-β (Aβ) and the proteins tau and α-synuclein form insoluble and highly ordered linear aggregates, known as amyloid fibrils. It is a challenge to study in real time the formation of the different molecular species on the pathway from monomeric to highly ordered fibrillar structures, but these studies are essential in order to explore the relationship between aggregate structure, toxic properties, and clinical symptoms further. In addition, robust assays of aggregation are vital in the search for, and investigation of, aggregation-inhibiting compounds that selectively interfere with individual molecular steps along the overall conversion pathway.

Although the morphology, size, and biological function of parent polypeptides are remarkably diverse, all amyloid fibrils share common structural properties. They consist of highly ordered β-strands that lie perpendicular to a common axis in a so-called cross-β arrangement.[2] The peptides are interconnected by an extensive network of hydrogen bonds aligned in parallel with the fibril axis. The hydrogen bond network stabilizes the amyloid fibrils and is thought to be the origin of their remarkable properties, such as high thermodynamic[3] and mechanical[4] stability. Recently, it has been found that amyloid-like structure is associated with an intrinsic fluorescent phenomenon that occurs in the visible range.[5–8]

In this paper, we show that this phenomenon can be used to track the aggregation of amyloid proteins noninvasively and quantitatively without any need for extrinsic labels. We validate the method against an established assay of protein aggregation and compare the increase in the intrinsic fluorescence emission intensity with the change in Thioflavin T (ThT) fluorescent signal under conditions that give highly reproducible fibril-growth kinetics. We quantify the conversion from the soluble into aggregated forms of two proteins in real time and use atomic force microscopy (AFM) and confocal imaging to probe the identity of the species giving rise to the observed fluorescence signatures. We also show that the assay is compatible with standard confocal microscope setups, so that both spatial (structural) and spectral information can be monitored simultaneously from fibril assemblies. The intrinsic fluorescence assay has an excellent dynamic range, similar to that of ThT fluorescence, but with the significant advantage that no extrinsic labels are required, neither is the presence of aromatic residues necessary;[9, 10] thus it can be applied to a wider range of amyloid proteins. Furthermore, we show that this assay permits us to study the interaction of small-molecule compounds with aggregates in situations in which the ThT assay fails. Using the effect of lacmoid on the aggregation of α-synuclein as an example, we show that the intrinsic fluorescence assay faithfully reports on amyloid growth, in contrast to ThT fluorescence, which is strongly affected by the presence of lacmoid.

## Results and Discussion

### Amyloid fibrils from different polypeptide sequences exhibit similar fluorescence properties in the visible range

**Figure 1:**
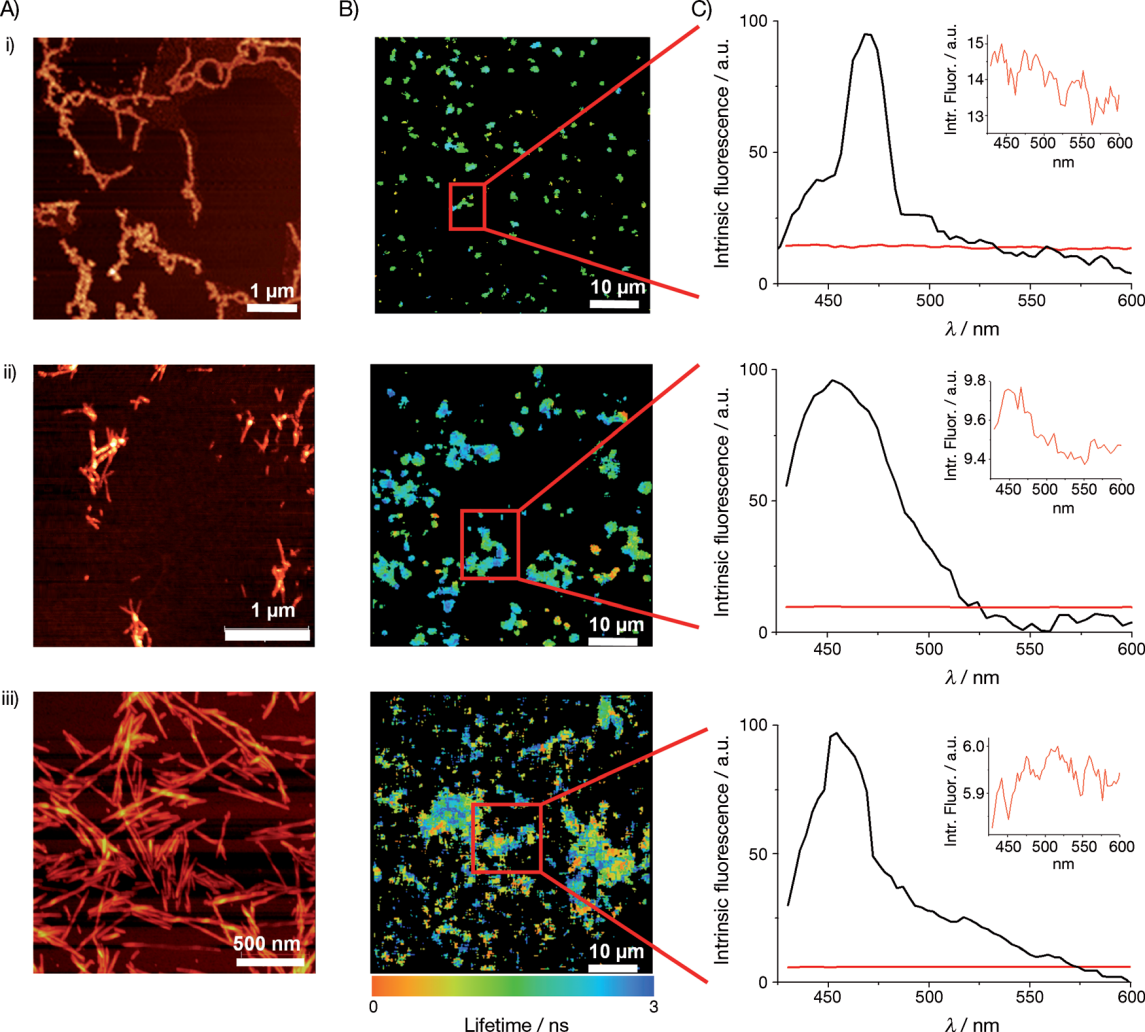
Intrinsic fluorescence in the visible range detected from three different amyloid fibrils formed in vitro. A) AFM images of fibrils formed from i) human tau K18, ii) human Aβ42 fibrils, and iii) human α-synuclein fibrils. B) Intrinsic fluorescence lifetime images of the same amyloid fibrils obtained by confocal microscopy. C) Black traces: emission spectra of the intrinsic fluorescence from specific aggregates as indicated in (B). Red traces: fluorescence signal from the corresponding monomeric protein (shown rescaled in the insets). The laser excitation wavelength was 405 nm.

We have performed detailed spectroscopic measurements on a series of amyloid fibrils, and all exhibited an intrinsic fluorescence, thus suggesting that this is a generic feature of the amyloid form of proteins. We used a purpose-built confocal microscope, equipped with spectral emission imaging and lifetime measurement capability,[11–13] to allow us to probe the fibril-assembly process directly and to measure the fluorescence properties of the aggregates, thereby showing that the emission originates from fibrillar species. The underlying morphology of the aggregates was also measured at higher resolution by AFM. Figure 1 shows an analysis of aggregates of the amyloid-β peptide (1–42) (Aβ42), the 129-residue tau protein isoform K18 (I260C/C291A/C322A variant) and the 140-residue and natively unfolded protein α-synuclein, which are associated with AD (Aβ42 and tau) and PD (α-synuclein). Figure 1A shows high-resolution AFM images of the fibril morphologies that reveal fibrillar structures for all three species, but also clear morphological differences between observed aggregates. Figure 1B shows the corresponding confocal fluorescence lifetime images of the same structures at a lower inherent resolution, but also reveals the presence of individual aggregate clusters. The color scale in Figure 1B reflects the fluorescence lifetimes of individual fibrils that were obtained by time-correlated single photon counting (TCSPC; see the Experimental Section). The lifetimes are in the 1 to 3 ns range, orders of magnitude larger than the excitation pulse length (ca. 10 ps). The corresponding fluorescence spectra are shown in Figure 1C. Clearly the fluorescence is Stokes shifted from the excitation wavelength (405 nm), with a peak centered at ∼460 nm and similar for all three species. The corresponding proteins in their monomeric form do not display any fluorescence signal, as is shown by the red traces in the insets. Fibrils could also be photobleached over extended periods of irradiation and furthermore exhibit two-photon excitation spectra similar to their one-photon counterparts shown here; the spectral properties are identical for solid-phase synthesized or recombinantly expressed amino acid sequences (see the Supporting Information). The similarities in the lifetime and spectral signatures between the three species are remarkable, given their differences in amino acid sequence and aggregate morphology. These findings substantiate the hypothesis made previously, that the intrinsic fluorescence phenomenon is a generic property of the cross β-sheet arrangement in amyloid fibrils,[5, 7] and is likely related to the extensive arrangement of hydrogen-bond networks running along the fibril axis.[6] Crucially, we also show that intrinsic fluorescence permits individual fibrils to be imaged microscopically without any use of extrinsic labels.

### Protein aggregation reactions can be spectroscopically monitored without the need for extrinsic labels

A key problem in the validation of a new assay of amyloid growth is the difficulty of obtaining standardized and calibrated aggregation conditions because the observed growth kinetics are the result of a complex interplay of underlying elementary reaction steps. Amyloid fibrils form through a nucleated growth process,[14] during which monomeric peptide slowly associates to form an oligomeric nucleus. This primary nucleation process is followed by an elongation phase in which the nucleus grows, typically by sequential incorporation of monomeric protein molecules, to form protofilaments and eventually mature fibrils. In addition, secondary pathways have been shown to play a significant role in the overall conversion of soluble peptide into aggregates.[15, 16] Such secondary processes include the fragmentation of fibrils[15] and monomer-dependent secondary nucleation, in which the fibrils themselves act to stimulate nucleation on their surfaces.[17] In the presence of preformed “seed” fibrils, the aggregation reaction can be considerably accelerated, and when the system is sufficiently heavily seeded, the only relevant molecular process is elongation, and thus the reaction follows exponential growth kinetics.[18] The resulting reduction in complexity of the aggregation pathway and the lack of dependence on stochastic primary nucleation reactions greatly improve the reproducibility of aggregation experiments and this is exploited in our work.

**Figure 2:**
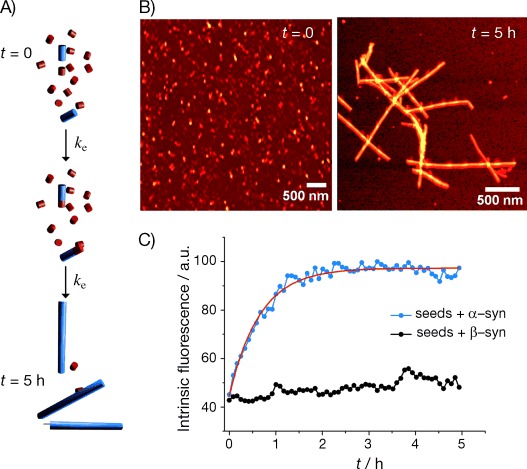
Kinetic assay of the seeded growth of α-synuclein. A) Simple schematic diagram illustrating the seeding process. Blue rods denote α-synuclein seeds, and red rods denote monomeric protein; *k*_e_=elongation rate constant. B) AFM images of sonicated seed fibrils and of the α-synuclein fibrils formed at the end of the reaction in the experiment shown in (C). C) Blue trace: Kinetic trace of the change in intrinsic fluorescence intensity over 5 h during seeded aggregation of α-synuclein. Seeds (7 μm) were incubated at 37 °C with α-synuclein monomeric protein (70 μm). Black trace: intrinsic fluorescence intensity over 5 h for α-synuclein seeds (7 μm) incubated with β-synuclein monomeric protein (70 μm); here no increase in the intrinsic fluorescence is observed, and hence no seeded growth. Red curve: exponential fit to the data.

In particular, we studied the seeded growth of amyloid fibrils of α-synuclein by using both the label-free intrinsic fluorescence and the ThT assays. The starting point was a sample containing preformed amyloid fibrils of the protein after sonication of preaggregated species to yield short seed fibrils (Figure 2A; see the Supporting Information). Protein monomer was added to this sample at a tenfold excess with respect to fibrils; the sample was then incubated at 37 °C for 5 h during which the intrinsic fluorescence intensity was recorded with a confocal microscope. The laser excitation wavelength was 405 nm, and the detection range was set to cover the 430 to 530 nm spectral range. Figure 2C illustrates the fluorescence intensity increase during aggregation, and Figure 2B shows AFM images that confirm the elongation of the fibrils. In the presence of a sufficient concentration of seed fibrils, the protein aggregation kinetics do not involve a lag phase and follow simple exponential behavior[18] due to monomer depletion. The observed increase in intrinsic fluorescence originates from the addition of soluble protein to the ends of seed aggregates, as depicted in the schematic of Figure 2A, and this leads to an overall increase of aggregate mass and length. The acquired data are well described by a single exponential function of the form *y*=*y*_0_+*A*_1_ exp(−(*t*/*t*_1_)) (red line in Figure 2C). As a control, we also incubated seed fibrils of α-synuclein with monomeric β-synuclein. The sequence of β-synuclein is related to that of α-synuclein, but the β form does not aggregate under the conditions used here[19] and is known not to be incorporated into α-synuclein fibrils.[20] As expected, the increase in intrinsic fluorescence is insignificant over a time period of several hours (Figure 2C, black curve). Furthermore, in an additional experiment (see the Supporting Information), we demonstrated that the intrinsic fluorescence tracks fibril growth in a linear, dose-dependent way.

**Figure 3:**
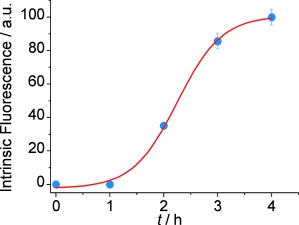
Nucleation and growth of tau K18 amyloid aggregates monitored by intrinsic fluorescence (blue dots). Red curve: sigmoidal fit to these points. Unlabeled human K18 tau monomers formed fibrils in vitro and were imaged on a confocal fluorescence microscope by using laser excitation at 405 nm. At *t*=0 heparin was added, and the ensuing aggregation reaction was observed for 4 h.

We also tested the capability of the assay to capture the full time course of aggregation in the absence of seed fibrils. We incubated a sample containing K18 tau monomers at 37 °C with heparin (see the Experimental Section) and recorded the time evolution of the intrinsic fluorescence intensity over several hours (Figure 3). The curve exhibits an initial lag phase until around *t*=1 h, after which a steep rise in signal is observed; this corresponds to the growth of fibrils. The rate slows down as the monomer is consumed in the conversion to amyloid fibrils; this yields overall the typical sigmoidal profile of a time course of amyloid formation.[16]

### Small molecules can cause interference with ThT fluorescence but do not affect the intrinsic amyloid fluorescence

**Figure 4:**
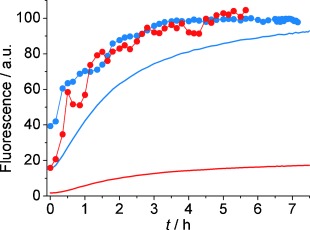
Kinetic traces of the change in intrinsic fluorescence intensity (•) and ThT fluorescence (—) during seeded aggregation of α-synuclein incubated at 37 °C with (red) and without (blue) added lacmoid (100 μm). In both cases, [fibril seed]=7 μm and [monomeric protein]=70 μm.

A detailed understanding of the protein aggregation reaction is crucial in order to explore new therapeutic strategies by rational means so as to find therapies against a range of debilitating human disorders. Traditional assays require extrinsic labels such as ThT,[21, 22] Congo Red (CR),[23] and 1-anilino-8-naphthalene sulfonate (ANS) dyes.[24] These systems are of great value, but in the search for small molecules labels can influence the kinetics and mechanisms of the aggregation process, as they can interfere with the binding of the inhibitor molecule or the inhibitor molecule can quench the fluorescence of the label.[25–27] We therefore tested the ability of our label-free intrinsic fluorescence assay to quantify amyloid growth in a situation in which experiments with ThT fluorescence are known to be challenging. Lacmoid is a small molecule that binds to amyloidogenic proteins and was previously thought to act as a potential inhibitor of aggregation. We therefore studied the aggregation of α-synuclein in the presence and absence of 100 μm lacmoid (Figure 4) and monitored the aggregation by using ThT and the intrinsic fluorescence assay. The intrinsic fluorescence is seen to rise in an almost identical fashion whether lacmoid is present (red points) or absent (blue points), and in agreement with earlier reports,[25] we thus verify that, at the stated concentration and in the presence of seed fibrils, lacmoid causes no inhibition of α-synuclein aggregation. The ThT fluorescence traces, however, vary significantly according to the presence (red line) or absence (blue line) of lacmoid. The observed dramatic decrease in fluorescence intensity (ca. fivefold) could be mistaken for an inhibitory action of lacmoid but is likely to be caused either by a quenching interaction between ThT and lacmoid, or by competition between ThT and lacmoid for free binding sites on the amyloid fibrils.

## Conclusions

In conclusion, we have demonstrated and characterized an all-optical kinetic assay of protein aggregation that is label-free and reproducible, and which can be used to study the kinetics of amyloid formation in real time. The assay is based on the intrinsic fluorescence associated with the amyloid form of polypeptides, an intriguing biophysical phenomenon that has recently been discovered and is stimulating much further biophysical investigation. The method eliminates the need for extrinsic labeling and thus excludes the risk of steric interactions and other perturbations during aggregation. Although the intrinsic fluorescence intensity is weaker than that of extrinsic fluorophores, our assay is simple to use (requiring only a confocal microscope), offers a similar dynamic range to traditional assays such as ThT, and reports quantitatively and reproducibly on amyloid fibrillar growth. We have demonstrated the potential of studying the interactions of small-molecule binding to amyloid fibrils through intrinsic fluorescence and shown that this method is more reliable than assays requiring extrinsic dye labels. Given its well-defined nature and straightforward application, we anticipate the widespread use of this method in the study of protein aggregation and in the search for small molecules that are potential therapeutic lead compounds.

## Experimental Section

**Materials:** All chemicals for the preparation of buffers, as well as the heparin and the lacmoid were purchased from Sigma–Aldrich. The “synthetic” human Aβ(1–42) (Bachem, Merseyside, UK) was prepared by sequential treatment with trifluoroacetic acid (TFA) and hexafluoroisopropanol (HFIP) to remove small aggregates and stored as a lyophilized film in Eppendorf tubes at −20 °C.[28] Samples were freshly thawed just before each set of experiments. Recombinant human Aβ(1–42) was expressed and purified as described previously,[29] except for the size-exclusion chromatography (SEC) step. The peptide was carefully divided into aliquots after the anion-exchange step. The tau construct K18 (I260C/C291A/C322A) was prepared as described in ref. [30]. Human α-synuclein was expressed and purified as described in ref. [31].

**Conversion of soluble polypeptides into fibrils:** Lyophilized, TFA- and HFIP-treated human Aβ(1–42) were dissolved in 1×PBS buffer (pH 7.4) at 50 μm peptide concentration in Eppendorf tube, and the solutions were incubated at 37 °C for 7 days. A lyophilized (from 1.5 mL, 10 mm Tris, pH 8.5, 125 mm NaCl, 1 mm EDTA) aliquot (≈15 nmol) of recombinant Aβ(1–42) was dissolved in deionized water (150 μL) and left to aggregate at room temperature overnight. K18 (I260C/C291A/C322A) tau (10 μm in 50 mm ammonium acetate buffer, pH 7.2) was incubated at 37 °C for 24 h, quiescent, in the presence of heparin (2.5 μm, MW3000, Sigma–Aldrich). All buffers were made with Milli-Q water and were filtered with 0.20 μm filters (Millipore) before use. Human α-synuclein (600 μm; in 20 mm phosphate buffer, pH 7.4) was incubated for 50 h at 45 °C under strong stirring with a Teflon-coated stir bar. The fibrils formed were diluted to 70 μm with PBS buffer and sonicated for 4 min with a probe sonicator (Bandelin, Solopuls HD 2070) at 10 % maximum power and with 30 % cycles.

**Thioflavin T kinetic assay:** Experiments with ThT were carried out in an Optima Fluostar platereader (BMG Labtech, Ortenburg, Germany). Samples were prepared that contained monomeric α-synuclein (70 or 35 μm), sonicated seed fibrils (7 or 3.5 μm) and Thioflavin T (100 μm). The experiments with lacmoid were performed by diluting a stock solution of lacmoid (10 mm) in DMSO 100-fold into the above solution of ThT, fibrillar, and monomeric α-synuclein. The control experiment in this case therefore contained 1 % (*v*/*v*) DMSO. We used half-area, low-binding, clear-bottom, 96-well plates (Corning). The experiments were performed under quiescent conditions so as to simulate as closely as possible the intrinsic fluorescence time-course measurements.

**Atomic force microscopy (AFM):** AFM images were acquired by using a VEECO Dimension 3100 atomic force microscope (Bruker) and a JPK Nanowizard (JPK, Cambridge, UK). The instruments were operated in tapping mode in air by using silicon cantilevers with a resonant frequency of 300 kHz, a spring constant of 40 N m^−1^ and a tip radius of 10 nm (RTESP, Bruker AXS). Images were collected at a scan rate of 1 Hz. Each fibrillar sample (5 μL) was deposited onto freshly cleaved mica surfaces for 2 h for adsorption. The samples were rinsed with Milli-Q water (5×200 μL) and left to dry completely in air before imaging.

**Confocal microscopy and spectral imaging:** Spatially resolved images of the intrinsic fluorescence of amyloid fibrils were obtained with a confocal microscope (Leica TCS SP5, Leica Microsystems), with a 405 nm diode laser as the excitation source and a 63-fold, 1.4 NA oil immersion objective. These experiments were performed on the same samples as imaged by AFM. An emission bandwidth of 20 nm was used for the spectral measurements. It was verified that photobleaching was insignificant during image and spectrum acquisition. In the kinetic experiments, the intrinsic fluorescence intensity at each time point was integrated over a defined confocal volume taken at ten different focal (*Z*) planes.

**Fluorescence lifetime imaging microscopy (FLIM):** All fluorescence lifetime images were recorded on a home-built confocal microscopy platform based on a confocal microscope scanning unit (Olympus FluoView 300) coupled with an Olympus IX70 inverted microscope frame. Details can be found in ref. [13].

## Notes

As a service to our authors and readers, this journal provides supporting information supplied by the authors. Such materials are peer reviewed and may be re-organized for online delivery, but are not copy-edited or typeset. Technical support issues arising from supporting information (rother than missing files) should be addressed to the authors.
