# Supplementary material for: A Label-Free, Quantitative Assay of Amyloid Fibril Growth Based on Intrinsic Fluorescence
Source: Chembiochem. 2013 Apr 16;14(7):846–50. doi: 10.1002/cbic.201300103 (PMC3790954; doi:10.1002/cbic.201300103)
Supplement: Supplementary file 1 [file cbic0014-0846-SD1.pdf]

CHEMBIOCHEM

Supporting Information

© Copyright Wiley-VCH Verlag GmbH & Co. KGaA, 69451 Weinheim, 2013

A Label-Free, Quantitative Assay of Amyloid Fibril Growth Based on Intrinsic Fluorescence

Dorothea Pinotsi,*[a] Alexander K. Buell,[b] Christopher M. Dobson,[b]
Gabriele S. Kaminski Schierle,[a] and Clemens F. Kaminski*[a]

cbic_201300103_sm_miscellaneous_information.pdf

Supplementary Figures

We have compared identical peptide sequences for the Aβ (1-42) peptide which were either synthesized from the solid phase or obtained by recombinant expression. We find that despite the very different origin and differing buffer conditions used, spectra and lifetimes are very similar for the two species, as depicted in Figure 1.

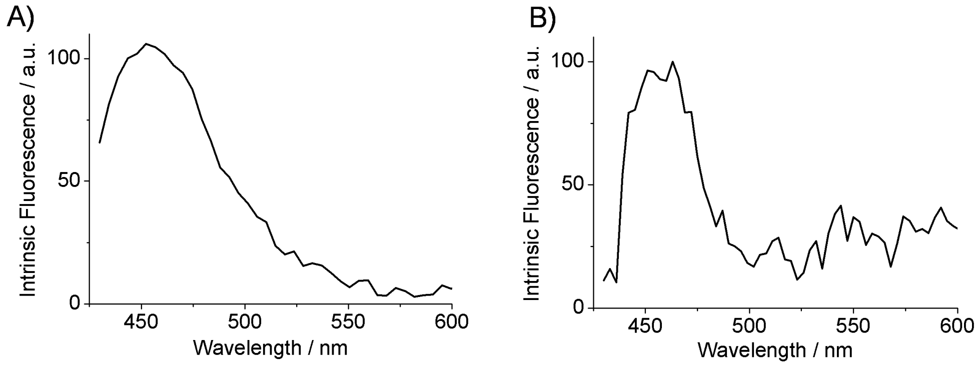

Figure 1: Synthetic and recombinant Aβ42 have similar spectroscopic properties. Intrinsic fluorescence emission spectra of A) 70μM synthetic and B) 50μM recombinant Aβ42 upon 405 nm laser excitation.

In a further experiment we established whether the intrinsic fluorescence is capable of tracking amyloid fibril growth quantitatively for different concentrations of monomer and different levels of seeding. Figure 2 shows traces obtained by ThT and intrinsic fluorescence experiments performed in parallel in the two different setups, on two samples whose initial protein and seed concentrations each differed by a factor of 2. Both assays yield similar fluorescence intensity increases, confirming that the intrinsic fluorescence tracks fibril growth in a linear fashion. The signal to noise ratio of the intrinsic fluorescence assay is lower compared to that of ThT, especially in the case of low seeding and monomer concentration (red dots). This is because the emission intensity of the intrinsic fluorescence is lower than that of extrinsic fluorophores and because of the reduced sample volume in the confocal setup, in contrast to the plate reader. Furthermore, the intrinsic fluorescence traces reflect a small time delay of the order of 10 min, required to get the instrument ready for image acquisition. Analyzing the time constants of the two different assays by fitting the data to an exponential growth function (black dashed lines), we note that the fits point towards a small, but significant difference between the measured rate constants in the intrinsic fluorescence and the ThT assay. This difference could be due to systematic differences in the two setups used for the assays, or more likely reflects the difference in the physical phenomena underlying the observed fluorescence signatures.

Another exciting avenue of the kinetic assay at hand is to use optical microscopy in total internal reflection illumination (TIRF) mode in order to monitor directly the growth of amyloid fibrils in a time lapse experiment, without the necessity of attaching a label. A preliminary experiment is depicted in Figure 3, where the intrinsic fluorescence of fully aggregated K18 tau is used to image the fibrils.

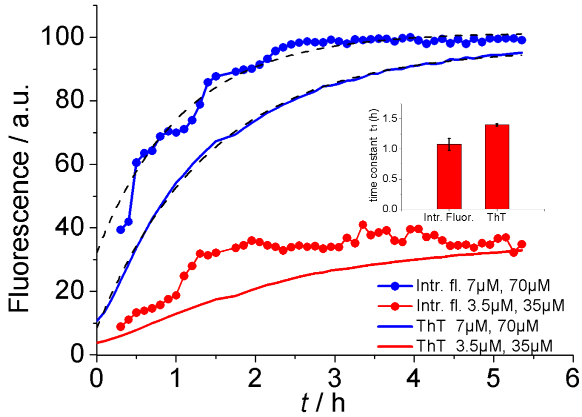

Figure 2: Comparison of kinetic traces of seeded aggregation of α-synuclein, using both ThT and also intrinsic fluorescence. The scaling with seed and monomer concentration was examined. Blue and red dots: intrinsic fluorescence, continuous lines: ThT fluorescence. Blue curves: 7 μM of seed concentration and 70 μM of monomeric α-synuclein protein. Red curves: 3.5 μM of seed concentration and 35 μM of monomeric α-synuclein protein. Black dashed lines: exponential fits to the data. Inset: time constant of the ThT and of the intrinsic fluorescence measurement obtained from the depicted exponential fits.

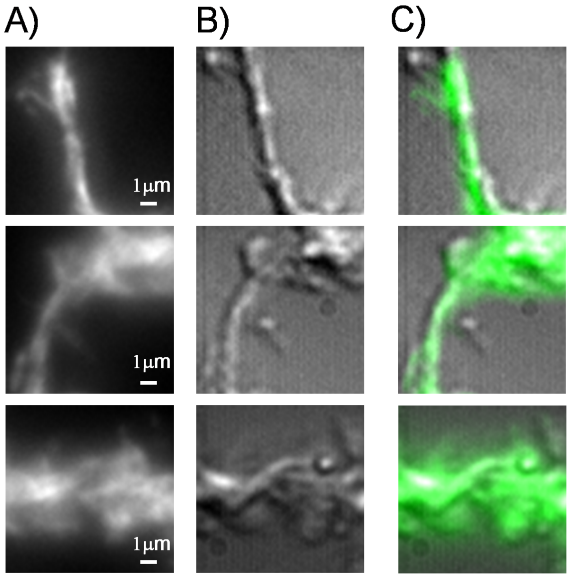

Figure 3: Intrinsic fluorescence imaged by a Total Internal Reflection (TIRF) microscope. A) Intrinsic fluorescence images of three different unlabeled tau K18 fibrils upon 405 nm excitation, obtained in TIRF illumination in a wide field microscope. B) Differential interference contrast (DIC) images of the corresponding fibrils in A) and C) overlay of the two images (green = intrinsic fluorescence).
